# Effects of letrozole supplementation on growth performance, plasma hormones, and plasma metabolites in weaned female lambs of Turpan black sheep

**DOI:** 10.3389/fvets.2026.1823444

**Published:** 2026-06-05

**Authors:** Tingting Li, Hui Chen, Jiaqi Liu, Hao Lu, Tingting Lu, Reyilaguli Reyimu, Xihu Wang, Jianjun Zhang, Shijie Li, Xiaojun Liu, Rui Xiao, Guodong Zhao, Kailun Yang

**Affiliations:** 1 Xinjiang Herbivore Nutrition Laboratory for Meat and Milk, College of Animal Science, Xinjiang Agricultural University, Urumqi, China; 2 Huishang Ecological Animal Husbandry Co., Ltd., Toksun County, Turpan, China; 3 Xinjiang Hutubi Breeding Cattle Farm Co., Ltd., Hutubi County, Changji, China; 4 Xinjiang Animal Embryo Engineering Technology Research Center, Hutubi County, Changji, China

**Keywords:** growth performance, letrozole, plasma differential metabolites, Turpan black sheep, weaned female lambs

## Abstract

This study investigates how adding letrozole (LE) to the diet affects the growth, blood hormones and metabolites, rumen fermentation, and gut microbes of weaned female Turpan Black sheep. The results could help guide strategies to enhance female growth and open new avenues for research. The study selected 60 weaned female lambs of the Turpan Black sheep breed, aged 2 months, with an average body weight of (19.93 ± 0.46) kg and in good health, which were randomly divided into the Con group (baseline diet), the 0.1 LE group (baseline diet + 0.10 mg/kg BW^−1^), and the 0.2 LE group (baseline diet + 0.20 mg/kg BW^−1^), with 20 lambs in each group. The pre-feeding period lasted 5 days, and the main feeding period lasted 75 days. The results showed that 0.2 LE group significantly improved total weight gain, body length and chest girth (*P* < 0.05), and markedly increased plasma GH and IGF-1 concentrations (*P* < 0.01). On days 15 and 75, plasma LE concentration in the 0.2 LE group was extremely higher than that in the 0.1 LE group (*P* < 0.01). Metabolomic analysis indicated that differential metabolites in the 0.1 LE group were mainly enriched in bile acid metabolism, with significant upregulation of estrone, taurocholic acid and fumaric acid (*P* < 0.05); while the 0.2 LE group presented altered cholesterol metabolism, accompanied by increased creatine and letrozole levels but decreased estrone and fumaric acid (*P* < 0.05). After 75 days of feeding, ruminal LE concentration in the 0.2 LE group was significantly higher than that in the 0.1 LE group (*P* < 0.05). All LE supplementation groups had higher rumen volatile fatty acid contents than the control group (*P* < 0.05), and the acetate-propionate ratio was higher in the 0.1 LE group relative to the 0.2 LE group (*P* < 0.05). LE treatment increased *Firmicutes* and decreased *Bacteroidetes* abundance. Within *Firmicutes*, the families *Lachnospiraceae* and *Eubacteriaceae* were higher in relative abundance. The LEfSe analysis showed that *Butyrivibrio fibrisolvens* was significantly enriched in the 0.1 LE group (*P* < 0.05), while *Oribacterium* was significantly enriched in the 0.2 LE group (*P* < 0.05). In conclusion, LE supplementation increased ruminal *Lachnospiraceae* and *Erysipelotrichaceae*, elevated butyrate, propionate and other VFAs. These products together with LE modulated bile acid/cholesterol metabolism and plasma hormones, supporting lamb growth. The optimal dose was 0.20 mg/kg BW^−1^.

## 1 Introduction

The Turpan Black Sheep is a local sheep breed raised for both meat and wool, primarily found in Toksu County, Turpan City. It has evolved through long-term natural selection and artificial breeding, with a reproduction rate of 97.09% (1). Zhang et al. (2) found that the sex ratio of Turpan Black sheep significantly influenced body conformation and meat production performance. They observed that yearling ewes weighed significantly less than rams, while ewes exhibited significantly higher fat deposition than rams. In production practice, most commercial sheep raised for meat are male lambs, while female lambs are primarily retained for breeding. Rapidly improving the growth performance of female lambs is also of great significance for the subsequent development of high-quality breeding ewes. Sex hormones, as important endogenous regulatory factors, influence both growth rate and reproductive performance in animals (3). Letrozole (LE) is a synthetic, third-generation, highly selective, non-steroidal aromatase inhibitor. Following oral administration, it has a half-life of 42 h and reaches peak serum concentrations within 1 h after rapid absorption in the gastrointestinal tract, effectively inhibiting estrogen synthesis (4). This mechanism reduces endogenous estrogen levels in the body, indirectly elevating circulating and intramuscular androgen concentrations (5). It primarily functions by competitively binding to the active site of cytochrome P_450_ aromatase, thereby blocking the conversion of androstenedione (AD) and testosterone (T) into estrone (E1) and estradiol (E2). However, hormonal regulation in animal organisms is a complex physiological process. Endogenous androgen levels undergo feedback regulation by estrogen, and elevated estrogen levels may suppress gonadotropin secretion via the hypothalamic-pituitary-gonadal axis, thereby affecting androgen synthesis and release (6).

As a non-steroidal aromatase inhibitor, LE has demonstrated its role in regulating sex hormone balance across multiple animal studies (7–9). Furthermore, early reproductive regulation in female ruminants has a significant impact on their reproductive performance throughout their lives. However, current physiological regulatory approaches for the pre-pubertal stage of weaned female lambs are limited. Therefore, this study used weaned female lambs of the Turpan Black sheep breed as the research subjects, to assess the effects of supplemental letrozole (LE) at different levels on the growth of weaned female Turpan Black sheep. Using non-targeted metabolomics to identify differentially expressed metabolites in plasma and 16S rRNA high-throughput sequencing to analyze rumen microbial community structure, we investigated the growth performance of letrozole-treated lambs and explored potential regulatory mechanisms. Although letrozole is not currently approved as a feed additive, the mechanistic findings of the present study provide theoretical insights for screening and designing safer early reproductive regulation strategies, such as plant-derived compounds targeting the aromatase pathway.

## 2 Materials and methods

### 2.1 Test site

The trial was conducted from February to May 2025 at Huishang Ecological Pastoral Industry Co., Ltd. in Toksun County, Turpan City, Xinjiang (coordinates 87°14′05″–89°11′08″E, 41°21′14″–43°18′11″N). The pre-feeding period lasted 5 days, and the main feeding period lasted 75 days.

### 2.2 Experimental design

**Figure 1:**
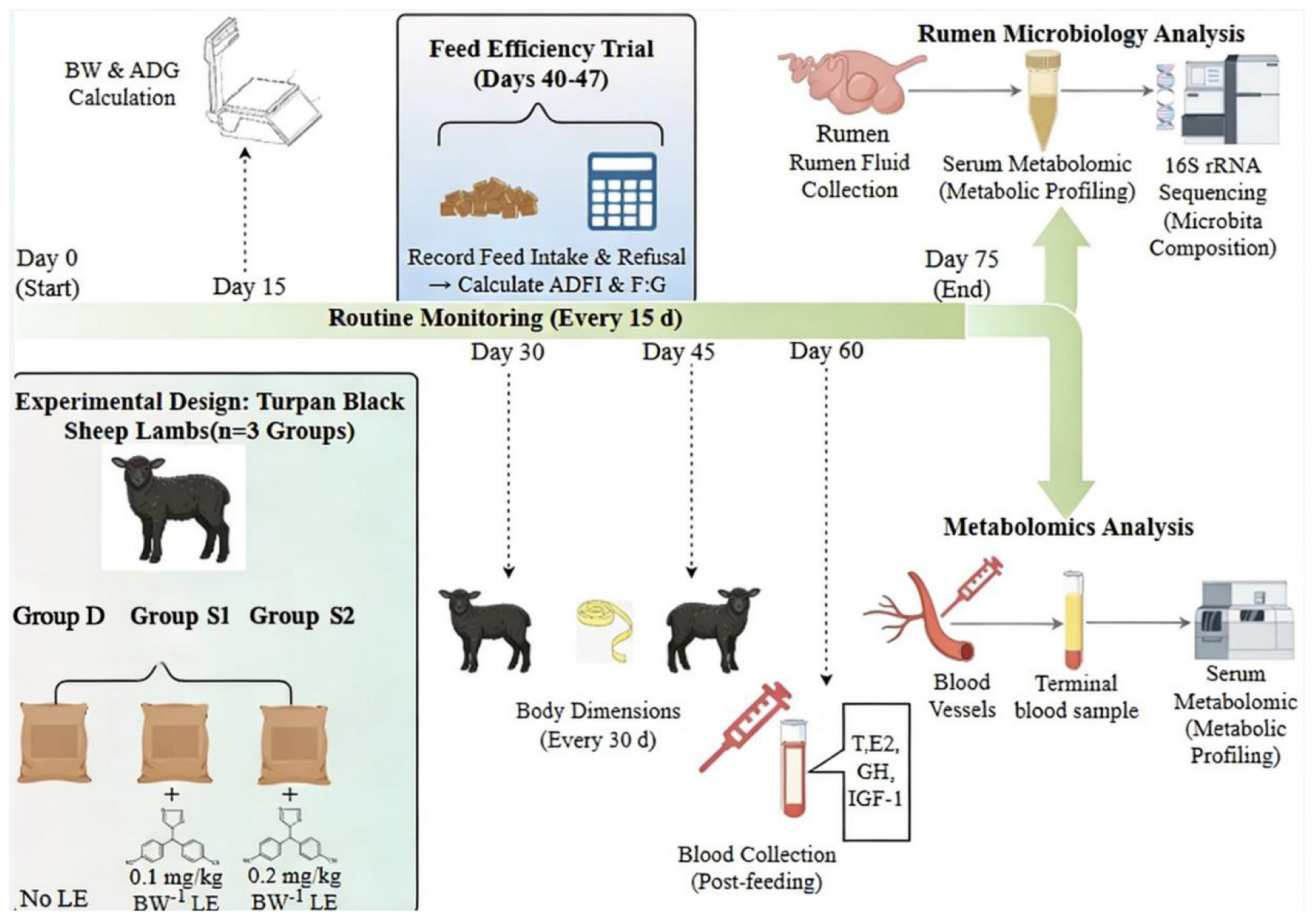
Experimental design flowchart.

The trial selected 60 weaned female lambs of the Turpan Black sheep breed, aged 2 months, with an average body weight of (19.93 ± 0.46) kg and in good health. They were randomly divided into 3 groups, with 20 lambs in each group. The groups were designated as follows: Con group (basal diet), 0.1 LE group (basal diet + 0.10 mg/kg LE), and 0.2 LE group (basal diet + 0.20 mg/kg LE). During the trial period, six lambs per group were randomly selected and fixed for subsequent sampling and index determination. Blood samples were collected at days 0, 30, and 75 for plasma hormone analysis, while rumen fluid samples were collected on day 75 for relative microbial abundance assessment in the rumen (Figure 1).

### 2.3 Feeding and management

All experimental lambs were ear-tagged, and the immunization schedule followed the farm's standard protocol. In addition to the basic diet, the Con group was supplemented with 2 g of dough; the 0.1 LE group and 0.2 LE group were supplemented with 0.10 and 0.20 mg/kg BW^−1^ of LE (purchased from Shandong Senlaixi Chemical Co., Ltd.), respectively, in addition to the Con group's diet. During the experiment, LE was evenly mixed into 2 g of dough and fed daily at 09:00 according to body weight. After restraining the lamb, dough was inserted into one corner of its mouth. The lamb's mouth was held closed with the hand to ensure complete swallowing of the dough, after which the lamb was released to move freely. The basic diet consisted of a total mixed ration (see Table 1 for the Basal Diet Formula and Nutrient Composition). Feed was provided at 10:00 and 16:30 each day, and all experimental lambs had free access to feed and water. The feed formulation was adjusted in accordance with (NYT816-2021 Nutritional Requirements for Meat Sheep). In addition, the main ingredients of lamb pellet concentrate (purchased from Xinjiang Jiaruile Biotechnology Co., Ltd.) consist primarily of corn, puffed soybeans, soybean meal, corn germ meal, bran, cottonseed meal, mineral elements and their complexes, vitamins and vitamin analogs, brewer's yeast (ruminant), amino acids, and antioxidants. It is fed at 11:30 a.m. and 5:30 p.m. daily, with a daily feed allowance of 200–300 g per bird. The concentrate-to-roughage ratio is 4:6.

**Table 1:** Basic Ration Composition and Nutrient Levels (DM basis) (%).

Ingredients	Content	Nutrient levels^②^	Content
Corn	52	CP	14.74
Soybean meal	7	EE	2.85
Corn germ meal	12.5	Ash	4.42
Wheat bran	17	NDF	18.95
Cotton seed meal	5	ADF	6.01
Premix^①^	5	Ca	0.63
Limestone powder	1.5	P	0.50
Total	100	ME/(MJ·kg^−1^)	10.66

Notes: ① Per kilogram of premix contains: vitamin A 150,000 IU, vitamin D_3_ 8,000 IU, vitamin E 1,000 IU, vitamin K_3_ 10 mg, thiamine 40 mg, riboflavin 40 mg, niacin 300 mg, pantothenic acid 150 mg, pyridoxine 40 mg, biotin 5 mg, folic acid 15 mg, cyanocobalamin 0.5 mg, choline 1,500 mg; copper 150 mg, zinc 600 mg, manganese 450 mg, iron 600 mg, iodine 15 mg, cobalt 5 mg, selenium 5 mg. ② Nutrient levels are determined measured values, and metabolizable energy is calculated value. Single measurement, no SD available. DM = Dry Matter; CP = Crude Protein; EE = Ether Extract; Ash = Crude Ash; NDF = Neutral Detergent Fiber; ADF = Acid Detergent Fiber; Ca = Calcium; P = Phosphorus.

### 2.4 Sample collection

#### 2.4.1 Feed sample collection

Diet samples were collected from the experimental lambs before and after feeding. For pre-feeding samples, five to eight random points were selected from the fresh diet to be fed, with 100 g collected from each point; Residual feed samples were collected at fixed intervals after the lambs had finished eating; the collected samples were placed in clean trays, thoroughly mixed, and 500–800 g of feed samples were taken, air-dried evenly, and then divided into portions and labeled with the date for use in determining feed moisture content and nutritional analysis.

#### 2.4.2 Growth performance data collection

Record the lambs' fasting body weight (Body Weight) on days 0, 15, 30, 45, 60, and 75 of the experiment, and calculate the average daily weight gain (ADG). Follow the method described by Li (10). Record daily feed intake and leftover feed, and calculate the average daily feed intake (ADFI) and feed conversion ratio (FCR) for each group.


$$\begin{array}{l} \text{ADG}=\left(\text{Final Body Weight}-\text{Initial Body weight}\right)\;/\text{ Trial Days}\\ \text{ADFI}=\text{Daily Feed Intaike }/\;\left(\text{Trial Days }\times \text{ Lamb}{\text{s}}^{\text{′}}\text{ Number}\right)\\ \text{ FCR}=\text{ADFI }/\text{ ADG} \end{array}$$

#### 2.4.3 Body measurement data collection

Body measurements were taken at days 0, 30, 60, and 75 of the trial. During measurement, lambs were led to level ground and allowed to stand naturally. Body height, Body length, and Chest Girth of Turpan Black sheep were measured and recorded.

#### 2.4.4 Blood sample collection

Before the experiment commenced, six lambs were randomly selected from each of the Con group, 0.1 LE group, and 0.2 LE group for scheduled jugular vein blood sampling. Blood samples were collected on days 0 (one day before supplemental feeding), 15, 30, 45, 60, and 75, with sampling performed at 2 h after feeding at each time point. In brief, 5 ml of blood was collected from the jugular vein and placed into sodium heparin anticoagulant tubes. Samples were centrifuged at 3,500 rpm for 15 min using a refrigerated high-speed centrifuge (Model 5427R, Eppendorf, Germany). The supernatant plasma was aspirated with a pipette, transferred into 2 ml cryovials, and stored at −80 °C until further analysis.

#### 2.4.5 Rumen fluid sample collection

Following the rumen fluid collection method described by Liu (11), at the end of the 75th day of the trial, six healthy female lambs of similar body weight were selected from each group. Two hours after supplementary feeding, 100 ml of rumen fluid was collected via the oral route using a negative-pressure extraction device. To avoid contamination by saliva, the first 50 ml of collected rumen fluid was discarded. The remaining fluid was filtered through four layers of medical gauze, aliquoted, and stored in liquid nitrogen for analysis.

### 2.5 Sample analysis

#### 2.5.1 Dietary nutrient content determination

The dry matter (DM) content of feed was determined using the 105 °C oven-drying method (GB/T 6435-2014); crude ash content was determined in accordance with (GB/T 6438-2025); and crude protein content was determined using the Kjeldahl method (AOAC 990.03). calcium (o-cresolphthalein colorimetric method; AOAC 985.01), phosphorus (ammonium vanadomolybdate colorimetric method; AOAC 985.01), and neutral detergent fiber (NDF) and acid detergent fiber (ADF) in the samples (AOAC 2002.04).

#### 2.5.2 Plasma hormone level assays

Plasma T (Catalog No. YJ703926), E2 (catalog no. YJ450381), Growth Hormone (GH; Catalog No. YJ710681), and IGF-1 (Insulin-like growth factor 1; Catalog No. YJ710602) were measured using enzyme-linked immunosorbent assay kits purchased from Shanghai Yuanju Technology Bio-Center.

#### 2.5.3 Plasma letrozole assay

The method for determining plasma LE concentration was adapted from Zarghi et al. (12) and performed using high-performance liquid chromatography (fluorescence detection). Transfer 400 μl of centrifuged plasma into a 1.5 ml Eppendorf tube. Add 600 μl of methanol (chromatography grade) to precipitate proteins. Mix thoroughly for 30 s. Incubate at 4 °C for 2 h, then centrifuge at 10,000 rpm for 10 min. Transfer the supernatant to a 2 ml brown sample vial for analysis. Mobile phase: acetonitrile-phosphate buffer (35:65, pH: 5.5). Assay conditions: using the XB-C18 reversed-phase column (4.6 × 100 mm, 5 μm), flow rate 1.0 mL/min; column temperature 22 °C; excitation wavelength 230 nm, emission wavelength 295 nm; linear elution; injection volume 20 μl.

#### 2.5.4 Plasma differential metabolite assay

Method as described in 2.4.4. Blood samples were collected from experimental lambs in each group 2 h after supplemental administration of LE on day 75 of the experiment. All plasma samples were thawed at room temperature. A 100 μl aliquot of thawed plasma was transferred to an Eppendorf tube, to which 400 μl of 80% methanol aqueous solution was added. The mixture was vortexed, then incubated on ice for 5 min. Centrifuge at 15,000 rpm, 4 °C for 15 min to obtain supernatant. Dilute a portion of the supernatant with mass spectrometry-grade water to a methanol concentration of 53%. Centrifuge at 15,000 × g, 4 °C for 15 min, collect the supernatant, and analyze by Liquid Chromatography-Mass Spectrometry System (LC-MS). This parameter was measured by Novogene Bioinformatics Technology Co., Ltd., Beijing.

### 2.6 Rumen assay

#### 2.6.1 Determination of rumen fermentation parameters

Determination of Ammoniacal Nitrogen (NH_3_-N) in rumen fluid followed Li (13) using the indophenol blue colorimetric method. Determination of Volatile Fatty Acids (VFA) in rumen fluid followed the method of Xu (14) using a Shimadzu GC-2010 gas chromatograph, calculating Total Volatile Fatty Acids (TVFA) concentration and the acetic acid to propionic acid ratio.

#### 2.6.2 Determination of letrozole content in the rumen

Method same as Section 2.5.3. Transfer 400 μl of filtered and centrifuged rumen supernatant into a 1.5 mL Eppendorf tube. Add 600 μl of methanol (chromatography grade) to precipitate proteins. Mix thoroughly for 30 s. Incubate at 4 °C for 2 h, then centrifuge at 10,000 rpm for 10 min. Transfer the supernatant to a 2 ml brown sample vial and prepare for instrument analysis. The measurement conditions are the same as in Section 2.5.3.

#### 2.6.3 Determination of rumen microbial diversity

Rumen 16S rRNA sequencing was performed by Novogene Bioinformatics Technology Co., Ltd. in Beijing. The universal bacterial primers (314F: 5′-CCTAYGGGRBGCASCAG-3′ and 806R: 5′-GGACTACNNGGGTATCTAAT-3′) were selected to amplify the bacterial 16S rRNA V3–V4 region via Polymerase Chain Reaction (PCR). The PCR products were electrophoresed on a 2% agarose gel. And the PCR products were purified using magnetic beads, followed by detection and recovery of the target band to generate a sequencing library. Genome sequencing was performed using the Illumina NovaSeq platform. Data analysis was conducted with Quantitative Insights Into Microbial Ecology (QIIME2) software, and visualization was achieved through the NovoMagic cloud platform.

### 2.7 Data statistics and analysis

#### 2.7.1 Statistical analysis of conventional indicators

Growth performance data, body measurements, and hormone data were preliminarily organized using Excel 2024. Statistical analysis was then performed using the mixed-model method in SAS 9.4 (SAS Institute Inc., Cary, North Carolina, USA). Results are presented as mean ± standard error. One-way analysis of variance (ANOVA) was performed on data such as rumen fermentation parameters using SPSS 27.0 statistical software, followed by Duncan's multiple range test for multiple comparisons; plasma LE metabolism data were preliminarily organized using Excel 2024 and then analyzed using an independent samples *t*-test in SPSS 27.0 statistical software; Data on LE metabolism in rumen fluid were preliminarily organized using Excel 2024 and then analyzed using an independent samples t-test with SPSS 27.0 statistical software. Results are expressed as (mean ± standard deviation). *P* < 0.05 was considered statistically significant, and a *P* < 0.01 was considered highly significant.

#### 2.7.2 Statistical analysis of metabolic indicators

Raw plasma metabolite data were processed using the metabolomics software Progenesis QI v3.0 (Waters Corporation, Milford, USA) for peak extraction, alignment, and identification. This yielded a data matrix containing retention times, peak areas, mass-to-charge ratios, and identification information for subsequent processing and bioinformatics analysis. The platform analysis was conducted by Beijing Novogene Bioinformatics Technology Co., Ltd.

#### 2.7.3 Statistical analysis of microbiological indicators

Microbial data analysis was conducted by Novogene Bioinformatics Technology Co., Ltd. in Beijing. The non-parametric Kruskal–Wallis test was used to analyze differences in rumen microbial diversity indices between groups and variations in relative abundance at the phylum, family, and genus levels. Metastats analysis was performed using R software (version 4.5.1) to identify species differences across various taxonomic levels.

#### 2.7.4 Correlation analysis

Pearson's correlation coefficient was used for the correlation analysis. Since this analysis was exploratory in nature, no multiple comparison correction (Bonferroni correction) was applied to the significance levels. All *P*-values are presented as raw values, and the potential risk of false positives was considered when interpreting the results. R software version 4.5.1 was used to analyze the correlations between reproductive hormones and rumen microbiota and plasma metabolites, and data visualization was performed using GraphPad Prism 9.0 and Adobe Illustrator 2025. Results are expressed as mean ± standard error. Differences were considered significant at ^*^P < 0.05, highly significant at ^**^P < 0.01, and extremely significant at ^***^P < 0.001.

## 3 Results

### 3.1 Effects of supplemental LE feeding on growth performance and weight gain in Turpan black lambs

As shown in Table 2, LE supplementation had no significant effect on lamb body weight for the group effect or the group × time interaction, but the time effect was highly significant (*P* < 0.01) for both the 0.1 LE and 0.2 LE groups compared with the Con group. In the 0.2 LE group, ADG was highly significantly higher under the time effect compared with the Con and 0.1 LE groups (*P* < 0.01), but no significant differences were found for the group effect or the Groups × Time interaction (*P* > 0.05). The 0.2 LE group showed significantly higher total weight gain than the Con group (*P* < 0.05), and a lower (but not significant) feed-to-gain ratio compared with the Con and 0.1 LE groups (*P* > 0.05).

**Table 2:** Effects of supplemental LE feeding on growth performance and weight gain in weaned female lambs of Turpan black sheep (*n* = 10; kg).

Item	Groups			*SEM*	*P*-value		
	Con group	0.1 LE group	0.2 LE group		Groups	Time	Groups × time
BW	19.64	20.14	20.41	0.53	0.06	< 0.01	0.61
ADG	0.15	0.16	0.18	0.01	0.09	< 0.01	0.62
TWG	11.09^b^	11.95^ab^	13.37^a^	0.45	0.05	–	–
ADFI	0.65	0.72	0.78	0.03	0.12	–	–
FCR	4.42	4.52	4.39	0.51	0.57	–	–

(1) BW, body weight; ADG, average body weight gain; TWG, total body weight gain; ADFI, average daily feed intake; FCR, feed conversion ratio. (2) Different lowercase letters on the same data point indicate significant differences (*P* < 0.05), different uppercase letters indicate highly significant differences (*P* < 0.01), and identical letters or no letters indicate no significant differences (*P* > 0.05). The same applies to the table below.

### 3.2 Effects of supplemental LE feeding on body measurements of Turpan black lambs

The Table 3 shown that LE supplementation had no significant effects on body height (group, time, or interaction, *P* > 0.05). In the 0.2 LE group, body length showed a highly significant time effect (*P* < 0.01) and a significant interaction effect (*P* < 0.05); chest girth showed highly significant time and interaction effects (*P* < 0.01).

**Table 3:** Effects of supplemental LE feeding on body measurements of weaned female lambs from Turpan black sheep (*n* = 10; cm).

Item	Groups			*SEM*	*P*-value		
	Con group	0.1 LE group	0.2 LE group		Groups	Time	Groups × time
Body height	61.50	61.79	61.68	0.93	0.93	< 0.01	0.91
Body length	63.96^b^	63.25^b^	65.50^a^	0.11	0.11	< 0.01	0.02
Chest girth	69.50^B^	69.82^B^	70.29^A^	0.80	0.80	< 0.01	0.01

Different lowercase letters on the same data point indicate significant differences (*P* < 0.05), different uppercase letters indicate highly significant differences (*P* < 0.01), and identical letters or no letters indicate no significant differences (*P* > 0.05). The same applies to the table below.

### 3.3 Effects of supplemental letrozole administration on plasma hormone levels in Turpan black lambs

As shown in Table 4, plasma T showed a highly significant time effect (*P* < 0.01) but no significant group or interaction effects (*P* > 0.05). Plasma E2 showed a highly significant time effect (*P* < 0.01), a significant group effect (*P* < 0.05), and no significant interaction (*P* > 0.05). For the 0.2 LE group, plasma GH and IGF-1 concentrations were highly significantly higher than those in the 0.1 LE and Con groups across all three effects (group, time, and interaction, *P* < 0.01).

**Table 4:** Effects of supplemental LE on plasma hormone levels in weaned female lambs of Turpan black sheep (*n* = 6).

Item	Groups			*SEM*	*P*-value		
	Con group	0.1 LE group	0.2 LE group		Groups	Time	Groups × time
T (pg/ml)	5.10	5.21	5.33	0.10	0.31	< 0.01	0.27
E2 (pg/ml)	28.46	28.73	26.97	0.47	0.02	< 0.01	0.11
GH (pg/ml)	1,517.7^B^	1,628.5^AB^	1,796.9^A^	26.3	< 0.01	< 0.01	< 0.01
IGF-1 (ng/ml)	3.91^C^	4.94^B^	5.86^A^	0.11	< 0.01	< 0.01	< 0.01

T, Testosterone; E2, Estradiol; GH, growth hormone; IGF-1, insulin-like growth factor 1.

Different lowercase letters on the same data point indicate significant differences (*P* < 0.05), different uppercase letters indicate highly significant differences (*P* < 0.01), and identical letters or no letters indicate no significant differences (*P* > 0.05).

### 3.4 Effects of LE supplementation on plasma LE content in Turpan black sheep lambs

As shown in Table 5, plasma LE content in 0.2 LE group was extremely significantly higher than that in 0.1 LE group on day 15 and day 75 of LE supplementation (*P* < 0.01). No significant differences were observed among all treatment groups on days 30, 45, and 60 of LE supplementation (*P* 0.05).

**Table 5:** Statistical analysis of differences in plasma metabolite concentrations following LE supplementation at different time points (*n* = 6).

Items	Groups		*P*-value
	0.1 LE group	0.2 LE group	
15d	30.89 ± 4.86^B^	47.86 ± 5.26^A^	< 0.01
30d	59.01 ± 5.24	59.57 ± 5.61	0.86
45d	40.98 ± 5.36	45.83 ± 5.88	0.17
60d	31.81 ± 4.90	36.81 ± 4.81	0.11
75d	38.71 ± 4.16^B^	48.91 ± 5.54^A^	< 0.01

Different lowercase letters on the same data point indicate significant differences (*P* < 0.05), different uppercase letters indicate highly significant differences (*P* < 0.01), and identical letters or no letters indicate no significant differences (*P* > 0.05).

### 3.5 Effects of supplemental letrozole on LE content and fermentation parameters in lamb rumen

As shown in Table 6, after 75 days of supplemental feeding, the rumen fluid LE content in 0.2 LE group was significantly higher than that in 0.1 LE group (*P* < 0.05). 0.1 LE group exhibited extremely significantly higher NH_3_-N content than Con group (*P* < 0.01), with no significant difference compared to 0.2 LE group (*P* > 0.05). 0.1 LE group had significantly higher acetate content than Con group (*P* < 0.05), with no significant difference compared to 0.2 LE group (*P* > 0.05). Propionic acid content in 0.2 LE group was significantly higher than that in Con group (*P* < 0.05), with no significant difference compared to 0.2 LE group (*P* > 0.05). Butyric acid content in both 0.1 LE group and 0.2 LE group was extremely significantly higher than that in Con group (*P* < 0.01). Isobutyric acid content in 0.1 LE group was extremely significantly higher than that in both Con group and 0.2 LE group (*P* < 0.01). 0.2 LE group exhibited extremely significantly higher valeric acid content than Con group (*P* < 0.01), with no significant difference compared to 0.1 LE group (*P* > 0.05). 0.1 LE group showed extremely significantly higher isovaleric acid content than 0.2 LE group (*P* < 0.01), while Con group had significantly higher isovaleric acid content than 0.2 LE group (*P* < 0.05). The total volatile fatty acid content in 0.1 LE group and 0.2 LE group was extremely significantly higher than that in Con group (*P* < 0.01). The acetic acid to propionic acid ratio in 0.1 LE group was significantly higher than that in 0.2 LE group (*P* < 0.05), with no significant difference compared to Con group (*P* > 0.05).

**Table 6:** Effects of supplemental Letrozole on LE content and fermentation parameters in lamb rumen (*n* = 6).

Items	Groups			*P*-value		
	Con group	0.1 LE group	0.2 LE group	Overall	Linear	Quadratic
Ruminal LE (ng/ml)	/	43.30 ± 3.9^8b^	49.12 ± 3.75^a^	0.03		
NH_3_-N (mg/dl)	19.05 ± 2.79^Bb^	25.25 ± 2.76^Aa^	22.81 ± 2.99^AaBb^	0.02	0.06	0.02
Acetic acid (mmol/L)	48.54 ± 4.95^b^	56.09 ± 4.39^a^	51.34 ± 4.85^ab^	0.04	0.32	0.20
Propionic acid (mmol/L)	20.58 ± 5.22^b^	22.07 ± 4.61^ab^	26.96 ± 2.28^a^	0.05	0.20	0.43
Butyric acid (mmol/L)	9.33 ± 1.62^Bb^	16.86 ± 2.33^Aa^	15.48 ± 2.69^Aa^	< 0.01	< 0.01	< 0.01
Isobutyric acid (mmol/L)	0.54 ± 0.08^Bb^	0.90 ± 0.07^Aa^	0.53 ± 0.10^Bb^	< 0.01	< 0.01	< 0.01
Valeric acid (mmol/L)	1.39 ± 0.54^Bb^	1.95 ± 0.39^ABab^	2.46 ± 0.47^Aa^	< 0.01	0.06	0.11
Isovaleric acid (mmol/L)	2.09 ± 0.31^ABa^	2.33 ± 0.49^Aa^	1.43 ± 0.48^Bb^	< 0.01	0.02	0.02
TVFA/ (mmol/L)	82.47 ± 8.65^Bb^	100.21 ± 7.93^Aa^	98.20 ± 8.50^Aa^	< 0.01	< 0.01	0.03
Acetic acid to propionic acid ratio	2.49^ab^	2.61^a^	1.91^b^	0.06	0.06	0.11

TVFA, total volatile fatty acids; different lowercase letters on the same data point indicate significant differences (*P* < 0.05), different uppercase letters indicate highly significant differences (*P* < 0.01), and identical letters or no letters indicate no significant differences (*P* > 0.05).

### 3.6 Effects of supplemental letrozole administration on differential plasma metabolites in lambs

#### 3.6.1 The PLS-DA model and ranking validation

**Figure 2:**
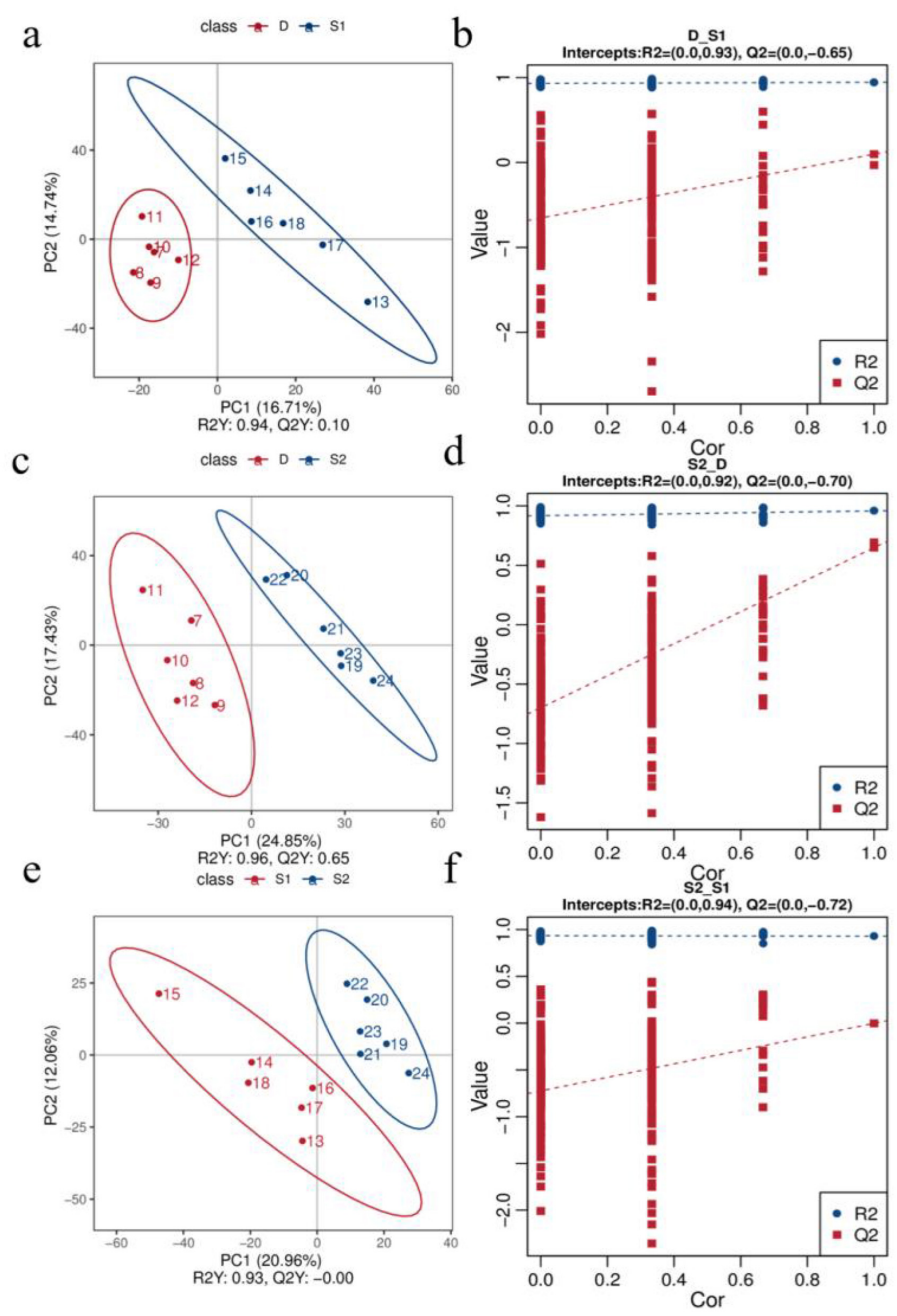
PLS-DA score scatter plot (**left**) and ranking verification plot (**right**). **(a, c, e)** PLS-DA score scatter plots for the comparison between Con .vs. 0.1 LE, Con .vs. 0.2 LE, and 0.1 LE .vs. 0.2 LE groups, respectively. **(b, d, f)** Permutation verification plots for the comparison between Con vs. 0.1 LE, Con vs. 0.2 LE, and 0.1 LE vs. 0.2 LE groups, respectively.

As shown in Figure 2, the results of the PLS-DA model established using the combined positive and negative ion mode indicate that all data points are statistically significant.

#### 3.6.2 Screening of plasma differential metabolites

**Figure 3:**
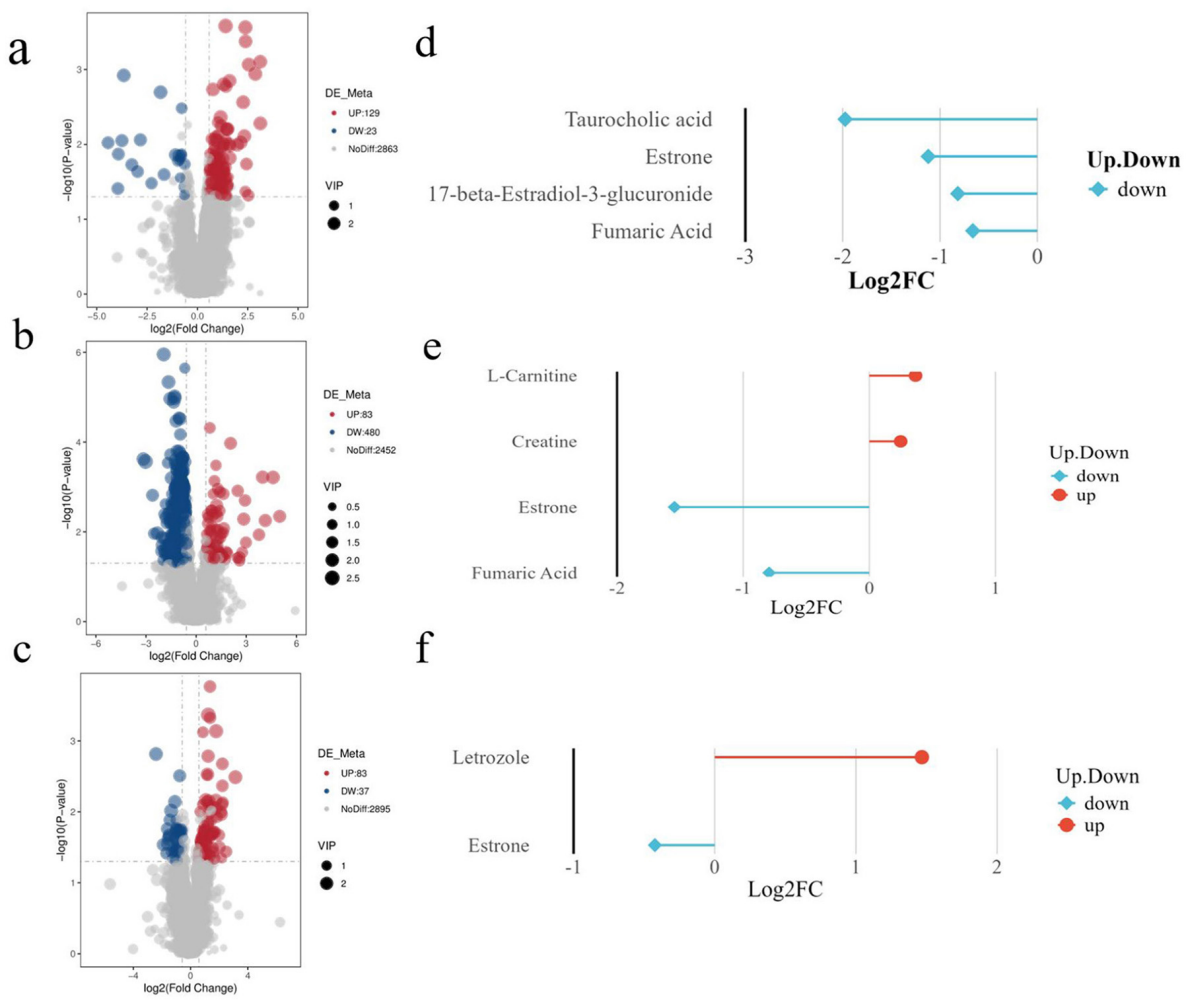
Differential metabolite screening volcano plots **(a–c)** and matchstick plots **(d–f)**. **(a, b, c)** Differential Metabolite Screening Volcano Map for the comparison between Con .vs. 0.1 LE, Con .vs. 0.2 LE, and 0.1 LE .vs. 0.2 LE groups, respectively. **(d, e, f)** Differential Metabolite Screening Matchstick Plot for the comparison between Con vs. 0.1 LE, Con vs. 0.2 LE, and 0.1 LE vs. 0.2 LE groups, respectively. In the volcano plot, red indicates differentially upregulated metabolites, blue indicates differentially downregulated metabolites, and gray indicates metabolites with no significant difference. The color of the matchstick bars distinguishes between upregulation (red) and downregulation (blue) of metabolites, while the bar length directly reflects the magnitude of the log2 (Fold Change) value–longer bars indicate greater fold changes. Additionally, the size of the dots represents the magnitude of the VIP value.

As shown in Figure 3a, among 3,015 detected plasma metabolites in lambs (Con group vs. 0.1 LE group), 152 showed significant changes (*P* < 0.05, 5.04% of total). Of these, 129 were upregulated (4.28% of total; 84.87% of significant) and 23 downregulated (0.76% of total; 15.13% of significant), while the remaining 2,863 metabolites were not significantly affected (*P* > 0.05). Following screening, the upregulated metabolites were identified as estrone, taurocholic acid, 17β-estradiol-3-β-D-glucuronide, fumaric acid, among others (Figure 3d).

As shown in Figure 3b, among 3,015 detected plasma metabolites (Con vs. 0.2 LE group), 563 showed significant changes (*P* < 0.05, 18.67% of total). Of these, 83 were upregulated (2.75% of total; 14.74% of significant) and 480 downregulated (15.92% of total; 85.26% of significant), while the remaining 2,452 metabolites were not significantly affected (*P* > 0.05). Among the differential metabolites screened between the Con group and the 0.2 LE group, upregulated metabolites included creatine and others, while downregulated metabolites included estrone, trans-2-hexadecenoyl-*L*-carnitine, fumaric acid, and others (Figure 3e).

As shown in Figure 3c, among 3,015 detected plasma metabolites (0.1 LE vs. 0.2 LE group), 120 showed significant changes (*P* < 0.05, 3.98% of total). Of these, 83 were upregulated (2.75% of total; 69.17% of significant) and 37 downregulated (1.23% of total; 30.83% of significant), while the remaining 2,895 metabolites were not significantly affected (*P* > 0.05). In the comparison between the 0.1 LE and 0.2 LE groups, the upregulated differential metabolite was letrozole, while the downregulated one was estrone (Figure 3f).

#### 3.6.3 KEGG enrichment pathway analysis of differentially metabolized plasma compounds

**Figure 4:**
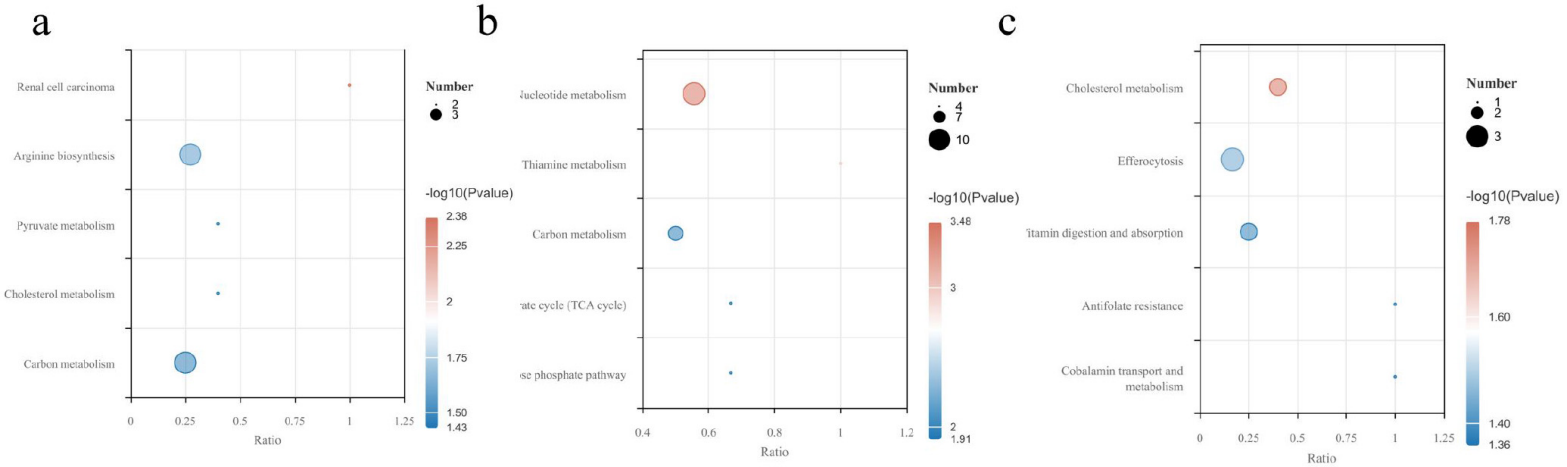
KEGG enrichment bubble plot. **(a)** Con vs. 0.1 LE group comparison; **(b)** Con vs. 0.2 LE group comparison; **(c)** 0.1 LE vs. 0.2 LE group comparison. The horizontal axis represents the enrichment ratio (Ratio), indicating the proportion of differentially expressed metabolites within the pathway. Bubble size corresponds to the number of differentially expressed metabolites in the pathway, while color intensity reflects the negative logarithm of the enrichment significance [–log_10_ (*P*-value)].

As shown in Figure 4a, the Kyoto Encyclopedia of Genes and Genomes (KEGG) results indicate that differentially expressed metabolites are significantly enriched in five key biological pathways, ranked in descending order of *P*-values as follows: Renal cell carcinoma, Carbon metabolism, Arginine biosynthesis, Citrate cycle/TCA cycle, Glucagon signaling pathway. The most prominent pathway was Renal cell carcinoma.

The KEGG pathway enrichment bubble plots for Con group and 0.2 LE group are shown in Figure 4b. The most significant pathways are Nucleotide metabolism and Thiamine metabolism.

Differentially enriched metabolites between 0.1 LE group and 0.2 LE group were significantly concentrated in 5 key metabolic pathways (as shown in Figure 4c), with the most prominent pathway being cholesterol metabolism.

### 3.7 Effects of supplemental LE on relative abundance of ruminant microbial communities in lambs

#### 3.7.1 Sequencing quality control

**Figure 5:**
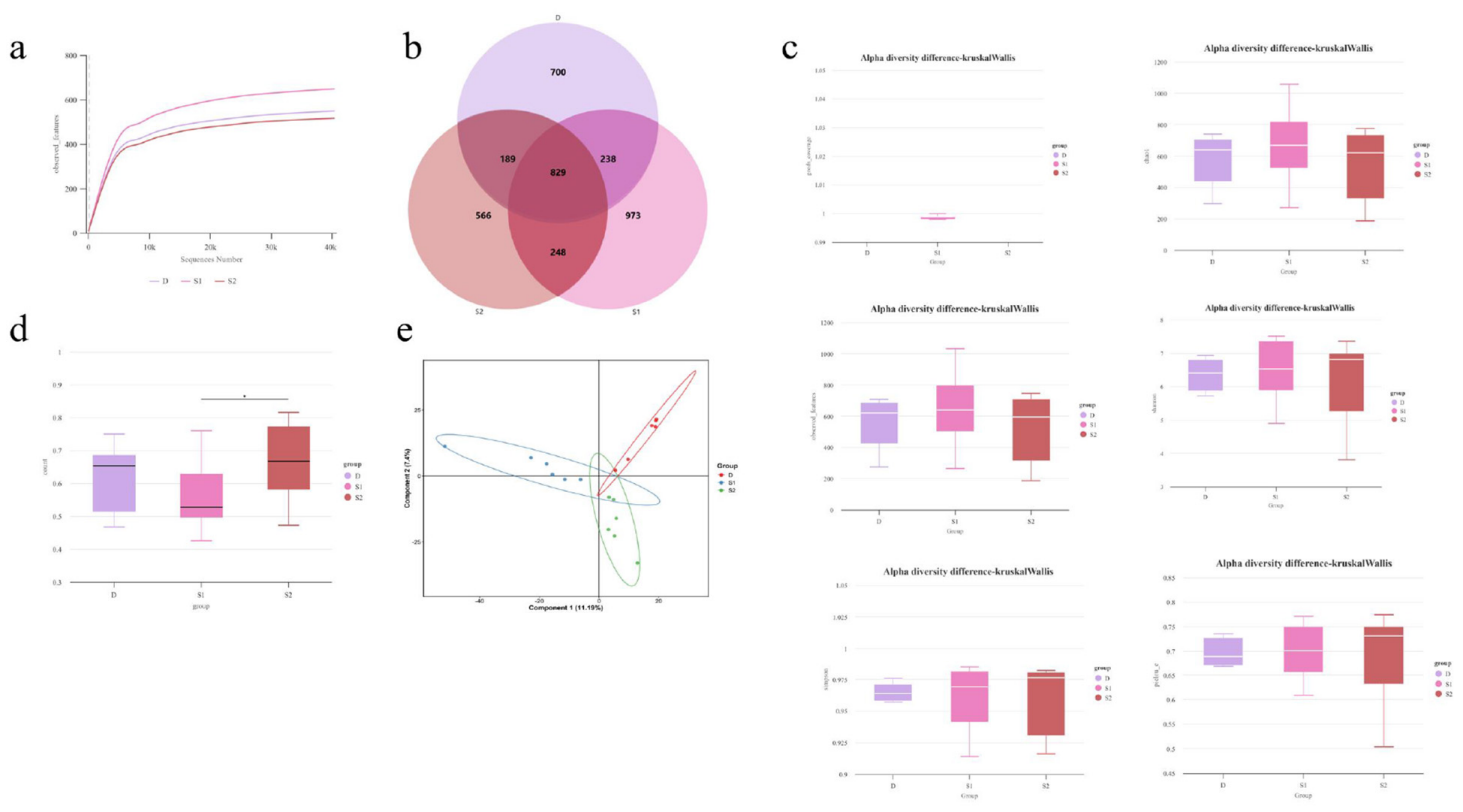
Effects of letrozole supplementation on rumen microbial diversity of lambs. D = Con group; S1 = 0.1 LE group; S2 = 0.2 LE group; **(a)** rarefaction curve of samples; **(b)** Venn analysis; **(c)** Alpha diversity of rumen microorganisms; **(d)** box plot of beta diversity indices across groups; **(e)** PLS-DA analysis plot.

As shown in Figure 5a, as the amount of sequencing data increases, the dilution curves for each sample group gradually flatten out, indicating that the current sequencing depth is sufficient to comprehensively cover the major taxonomic groups within the microbial communities of the samples. This confirms that the sequencing data is adequate to support the reliability of subsequent analyses.

#### 3.7.2 Venn analysis of rumen microbiota

As shown in Figure 5b, a total of 3,743 Operational Taxonomic Units (OTUs) were measured in this experiment. Among these, a number of 829 OTUs were shared across all three groups, accounting for 22.15% of the total OTUs. Con group and 0.1 LE group shared 1,067 OTUs, representing 28.51% of the total OTUs. Con group and 0.2 LE group shared 1,018 OTUs, accounting for 27.20% of the total; 0.1 LE group and 0.2 LE group shared 1,077 OTUs, representing 28.77%. The number of OTUs unique to Con group, 0.1 LE group, and Group II was 700, 973, and 566, respectively, accounting for 18.70%, 25.99%, and 15.12% of the total OTUs.

#### 3.7.3 Alpha diversity analysis

As shown in Figure 5c, based on the Kruskal–Wallis test, no significant differences were observed among Con group, 0.1 LE group, and 0.2 LE group for any of the indices: goods_coverage, Chao1, observed_features, Shannon, Simpson, and Pielou_e (*P* > 0.05). However, overall, the sample detection depth (goods_coverage) across all groups reached 0.99; the Shannon and Simpson indices adequately reflected the richness and diversity of the sample communities; and the Pielou_e index values were close to 1, indicating relatively even species distribution. In summary, the results from the alpha diversity analysis can be utilized for subsequent analyses.

#### 3.7.4 Beta diversity analysis

Analysis of intergroup differences in beta diversity indices using the Kruskal–Wallis test revealed significant differences in the distribution of these indices among the three groups (Figure 5d). The beta diversity index was significantly higher in 0.2 LE group than in 0.1 LE group (*P* < 0.05), suggesting marked differences in microbial community structure between the two groups.

#### 3.7.5 PLS-DA analysis

This figure shows the results of partial least squares discriminant analysis (PLS-DA) of the ruminal microbial community structure in the Con group, the 0.1 LE group, and the 0.2 LE group following the supplementation of letrozole. The horizontal axis of the figure represents the first principal component (COMP1), which accounts for 11.19% of the total variance; the vertical axis represents the second principal component (COMP2), which accounts for 7.4% of the total variance. As shown in the Figure 5e, samples from different groups exhibit a certain degree of separation in the two-dimensional PLS-DA space, indicating differences in ruminal fluid microbial community structure among the groups.

#### 3.7.6 Effects of supplemental letrozole on relative abundance of microorganisms in lamb rumen

**Figure 6:**
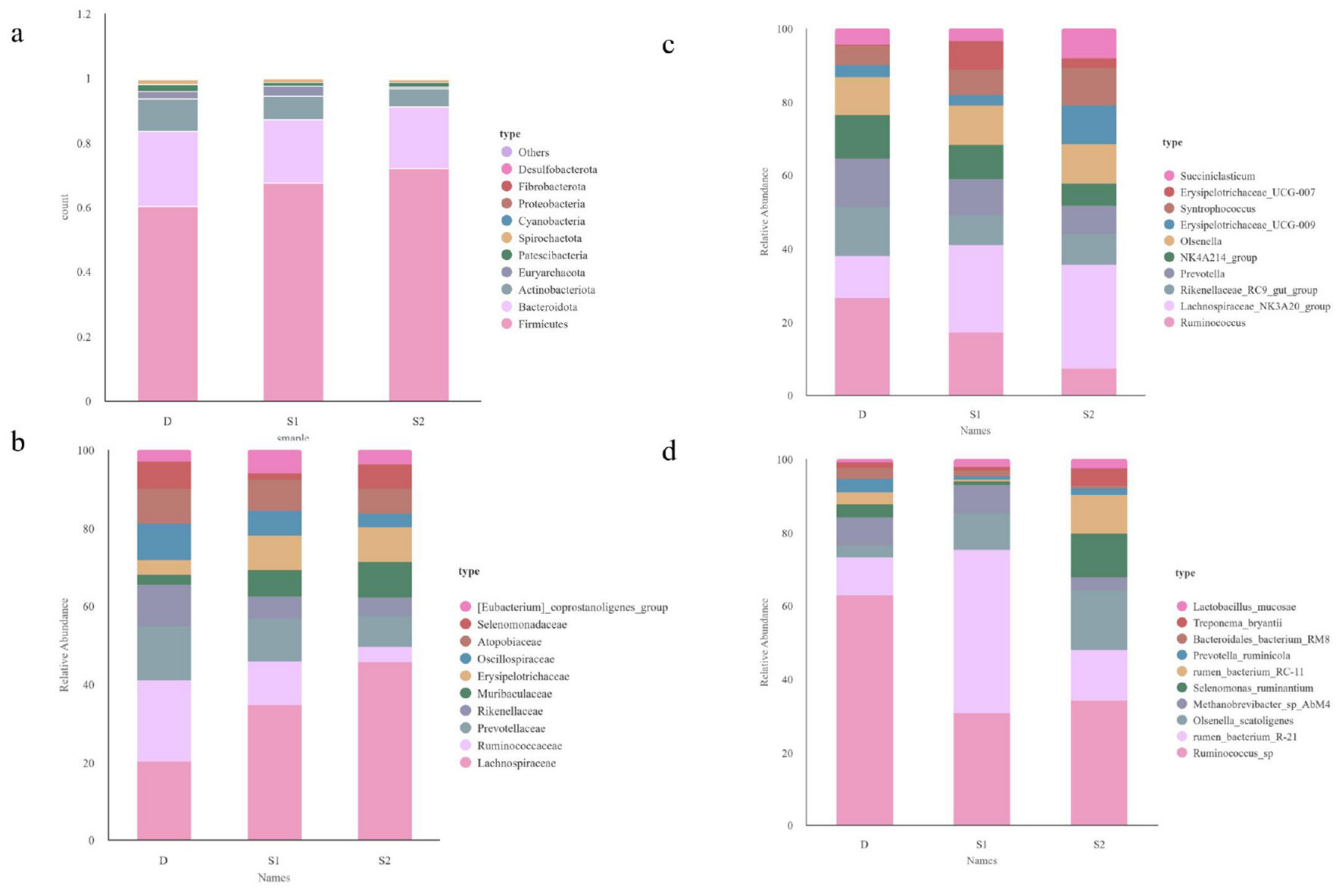
Bar chart of relative abundance of rumen microorganisms. **(a)** Phylum level; **(b)** family level; **(c)** genus level; **(d)** species level.

The relative abundance of rumen microbial phyla for Con group, 0.1 LE group, and 0.2 LE group is shown in Figure 6a. The top 10 phyla identified were Firmicutes (60.22%; 67.41%; 71.81%), Bacteroidetes (23.06%; 19.78%; 19.14%), Actinobacteria (10.26%; 7.05%; 5.51%), Euryarchaeota (2.19%; 3.09%; 0.71%), Patescibacteria (2.13%; 1.24%; 1.30%), Spirochaetota (1.52%; 1.12%; 1.05%), Cyanobacteria (0.31%; 0.13%; 0.09%), Proteobacteria (0.06%; 0.04%; 0.10%), Fibrobacterota (0%; 0%; 0.03%), and Desulfobacterota (0.01%; 0.03%; 0.06%). Among these, Firmicutes and Bacteroidetes were dominant phyla in all groups, with 0.2 LE group exhibiting higher abundance than 0.1 LE group and Con group. Actinobacteria and Proteobacteria were dominant in Con group, while Proteobacteria, Fibrobacterota, and Desulfobacterota were dominant in 0.2 LE group.

The relative abundance of rumen microbial families in Con group, 0.1 LE group, and 0.2 LE group is shown in Figure 6b. The top 10 families identified through screening were Lachnospiraceae (15.15%; 27.53%; 35.88%), Ruminococcaceae (15.65%; 9.10%; 3.28%), Erysipelotrichaceae (2.84%; 7.08%; 7.03%), Muribaculaceae (1.79%; 5.36%; 7.14%), Prevotellaceae (10.40%; 8.63%; 6.14%), Selenomonadaceae (5.22%; 1.36%; 4.97%), Oscillospiraceae (7.05%; 5.11%; 2.85%), Rikenellaceae (8.23%; 4.54%; 3.71%), Atopobiaceae (6.81%; 6.17%; 5.00%), Christensenellaceae (2.11%; 4.00%; 1.59%). Among these, Ruminococcaceae, Prevotellaceae, Oscillospiraceae, and Rikenellaceae were the dominant bacterial families in Con group; Atopobiaceae and Christensenellaceae were dominant in 0.1 LE group; and Lachnospiraceae, Muribaculaceae, Erysipelotrichaceae, and Selenomonadaceae were dominant in 0.2 LE group.

The relative abundance of rumen microbial genera in Con group, 0.1 LE group, and 0.2 LE group is shown in Figure 6c. The top 10 genera identified through screening were: *Ruminococcus* (15.38%; 9.07%; 3.18%), *Lachnospiraceae NK3A20_group* (6.70%; 12.63%; 12.54%), *Erysipelotrichaceae UCG-007* (0.00%; 4.18%; 1.11%), *Erysipelotrichaceae UCG-009* (1.86%; 1.47%; 4.67%), *Prevotella* (7.64%; 5.17%; 3.41%), *NK4A214_group* (6.94%; 4.88%; 2.68%), *Rikenellaceae_RC9_gut_group* (7.97%; 4.45%; 3.64%), *Olsenella* (6.03%; 5.81%; 4.74%), *Syntrophococcus* (3.19%; 3.69%; 4.53%), *Succiniclasticum* (2.56%; 1.80%; 3.61%). Among these, the dominant genera in Con group were *Ruminococcus, Prevotella, NK4A214_group, Rikenellaceae_RC9_gut_group*, and *Olsenella*; 0.1 LE group dominant genera included *Lachnospiraceae NK3A20_group* and *Erysipelotrichaceae UCG-009*; 0.2 LE group dominant genera comprised *Lachnospiraceae NK3A20_group, Erysipelotrichaceae UCG-009, Syntrophococcus*, and *Succiniclasticum*.

The relative abundance of rumen microbial species in Con group, 0.1 LE group, and 0.2 LE group is shown in Figure 6d. The top 10 bacterial species identified were: *rumen_bacterium_R-21* (1.23%; 5.30%; 0.81%), *Ruminococcus*_sp (7.56%; 3.65%; 2.02%), *Methanobrevibacter*_sp_*AbM4* (0.92%; 0.92%; 0.21%), *Olsenella_scatoligenes* (0.40%; 1.12%; 0.96%), *Selenomonas_ruminantium* (0.41%; 0.12%; 0.70%), *rumen_bacterium_RC-11* (0.40%; 0.05%; 0.63%), *Prevotella_ruminicola* (0.46%; 0.10%; 0.11%), *Treponema_bryantii* (0.16%; 0.11%; 0.28%), *Bacteroidales_bacterium_RM8* (0.37%; 0.20%; 0.04%), *Lachnospiraceae_bacterium_DJF_B223* (0.00%; 0.00%; 0.20%). Among these, the dominant species in Con group and 0.1 LE group were *rumen_bacterium_R-21* and *Ruminococcus*_sp, respectively. 0.2 LE group was primarily dominated by *Selenomonas_ruminantium* and *Methanobrevibacter*_sp_*AbM4*, while also containing higher proportions of *Treponema_bryantii* and *Lachnospiraceae_bacterium_DJF_B223*.

#### 3.7.7 Statistical tests

##### 3.7.7.1 LEfSe analysis

**Figure 7:**
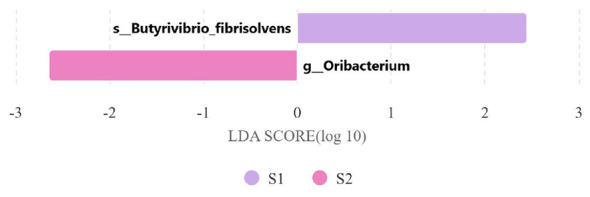
LEfSe analysis. S1 = 0.1 LE group; S2 = 0.2 LE group.

Further analysis using Linear discriminant analysis Effect Size (LEfSe) and based on the effect pattern LDA=2 identified microorganisms with significantly different relative abundances among the three groups, as shown in Figure 7. 0.1 LE group exhibited significant enrichment of *s_Butyrivibrio fibrisolvens* (*P* < 0.05); 0.2 LE group showed significant enrichment of *g_Oribacterium* (*P* < 0.05).

##### 3.7.7.2 Analysis of differences among multiple groups at the level

**Figure 8:**
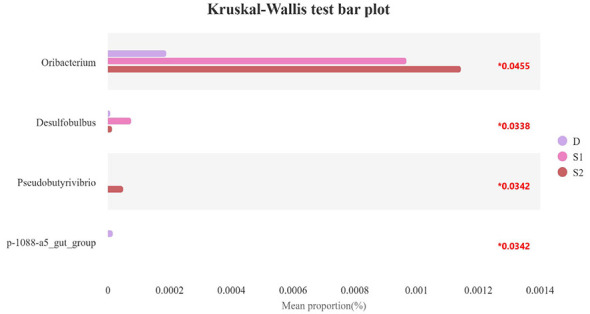
Horizontal multi-Con group difference analysis. The results of the multiple-Con group difference analysis were obtained using Dunn's *post-hoc* test with Bonferroni multiple comparison correction. The values on the right represent *P*-values, where *indicates *P* < 0.05, **indicates *P* < 0.01, and ***indicates *P* < 0.001. D = Group D; S1 = Group S1; S2 = Group S2.

The results of the genus-level interCon group differential analysis are shown in Figure 8. Comparative analysis revealed that the genera exhibiting significant differences were *Oribacterium, Desulfobulbus, Pseudobutyrivibrio*, and *p-1088-a5_gut_group* (*P* < 0.05). Among these, 0.2 LE group *Oribacterium* and *Pseudobutyrivibrio* were significantly higher than Con group and 0.1 LE group; 0.1 LE group *Desulfobulbus* was significantly higher than Con group and 0.1 LE group; Con group *p-1088-a5_gut_group* was significantly higher than 0.1 LE group and 0.2 LE group (*P* < 0.05).

### 3.8 Correlation analysis

#### 3.8.1 Correlation analysis between horizontal rumen microbiota and plasma metabolites

**Figure 9:**
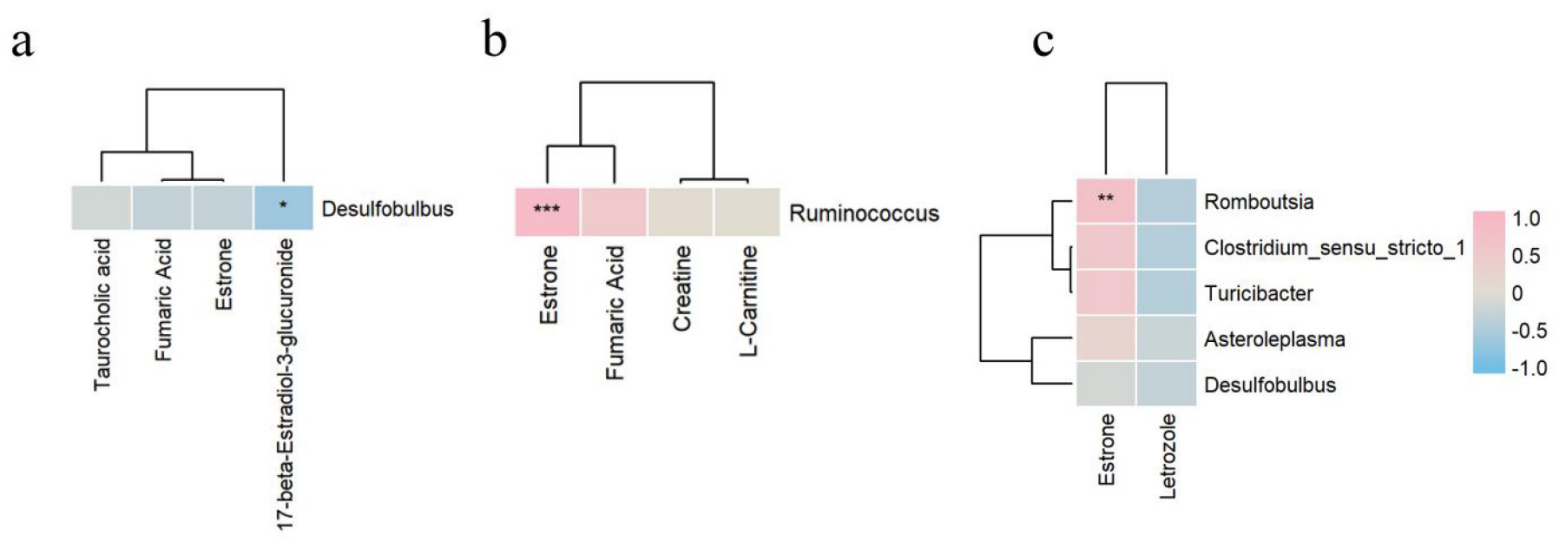
Correlation analysis between rumen microorganisms and plasma metabolites. **(a)** Shows Con group metabolites with differences between urine and plasma levels; **(b)** Shows 0.1 LE group metabolites with differences between urine and plasma levels; **(c)** Shows Con group metabolites with differences between urine and plasma levels. The values on the right represent *P*-values, where *indicates *P* < 0.05, **indicates *P* < 0.01, and ***indicates *P* < 0.001.

As shown in Figure 9a, the Pearson correlation analysis of the differences in metabolites in screened plasma and rumen microbiota at the genus level between the Con group and the 0.1 LE group. The results revealed that the genus Desulfobulbus showed a significant negative correlation with 17β-estradiol-3-glucuronide (*P* < 0.05), and a negative trend with estrone, taurocholic acid, and fumaric acid (*P* > 0.05). When comparing the Con group with 0.2 LE group, Ruminococcus showed a highly significant positive correlation with estrone (*P* < 0.001), and showed a positive correlation trend with fumaric acid (*P* > 0.05), while no significant differences were observed for creatine and trans-2-hexadecenoyl-L-carnitine (*P* > 0.05; see Figure 9b). Comparing 0.1 LE group with 0.2 LE group, estrone showed a significant positive correlation with Romboutsia (*P* < 0.01). Letrozole showed a negative correlation trend with Asteroleplasma, Desulfobulbus, Clostridium sensu stricto 1, Romboutsia, and Turicibacter (*P* > 0.05; as shown in Figure 9c).

#### 3.8.2 Correlation analysis between the relative abundance of horizontal rumen microorganisms and rumen fermentation parameters

**Figure 10:**
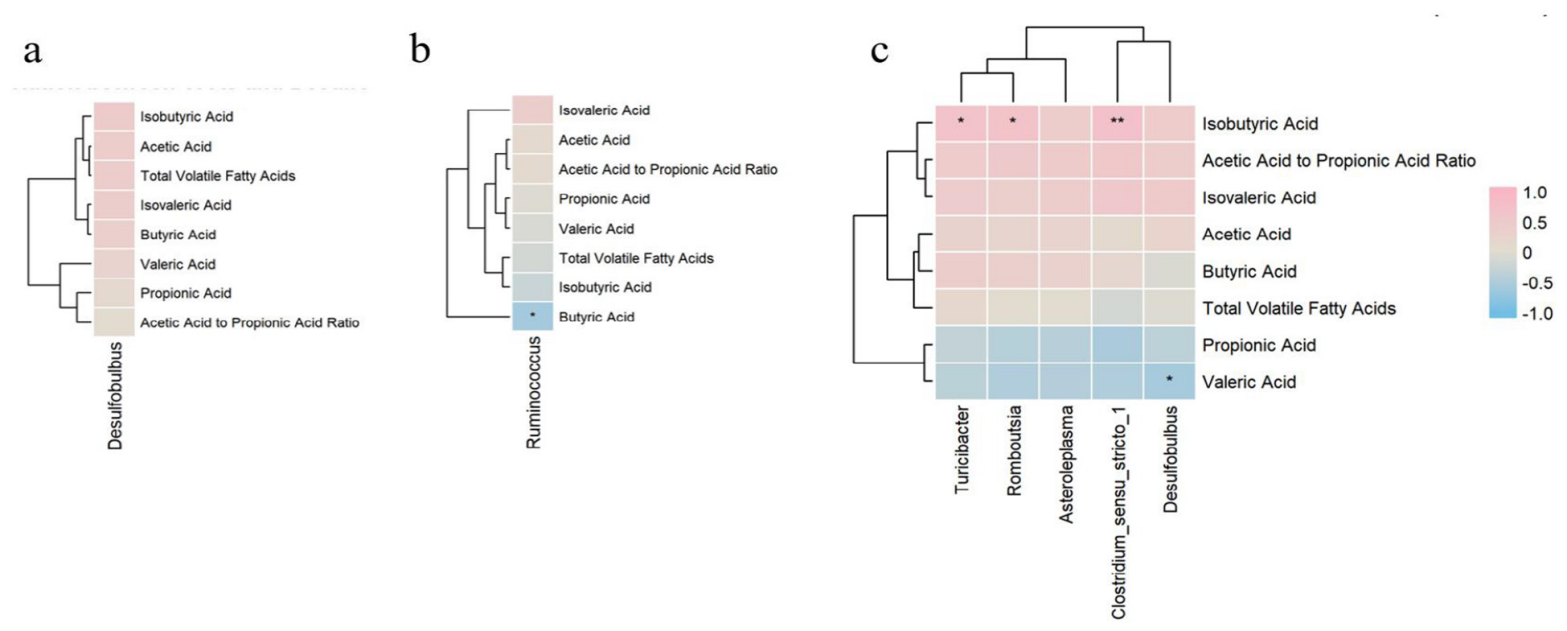
Heatmap of the correlation between rumen microorganisms and rumen fermentation parameters in cattle. **(a)** Shows Con group metabolites with differences between urine and plasma levels; **(b)** Shows 0.1 LE group metabolites with differences between urine and plasma levels; **(c)** Shows Con group metabolites with differences between urine and plasma levels. The values on the right represent *P*-values, where *indicates *P* < 0.05, **indicates *P* < 0.01, and ***indicates *P* < 0.001.

The results of the correlation analysis between the relative abundance of rumen microorganisms at the genus level and rumen fermentation parameters across groups, using Pearson correlation analysis, are shown in Figure 10. When comparing the Con group and the 0.1 LE group, Desulfobulbus showed a positive correlation trend with acetic acid, propionic acid, and butyric acid (*P* > 0.05; see Figure 10a). When comparing the Con group with the 0.1 LE group, the genus Ruminococcus showed a significant negative correlation with butyric acid (*P* < 0.05) and a positive trend with isovaleric acid (*P* > 0.05; see Figure 10b) . Comparing the 0.1 LE group and the 0.2 LE group, isobutyric acid showed a highly significant positive correlation with Clostridium sensu stricto 1 (*P* < 0.01) and a significant positive correlation with Romboutsia and Turicibacter (*P* < 0.05); valeric acid showed a significant negative correlation with Desulfobulbus (*P* < 0.05; see Figure 10c).

#### 3.8.3 Correlation analysis of the effects of LE supplementation on plasma metabolites and hormones

**Figure 11:**
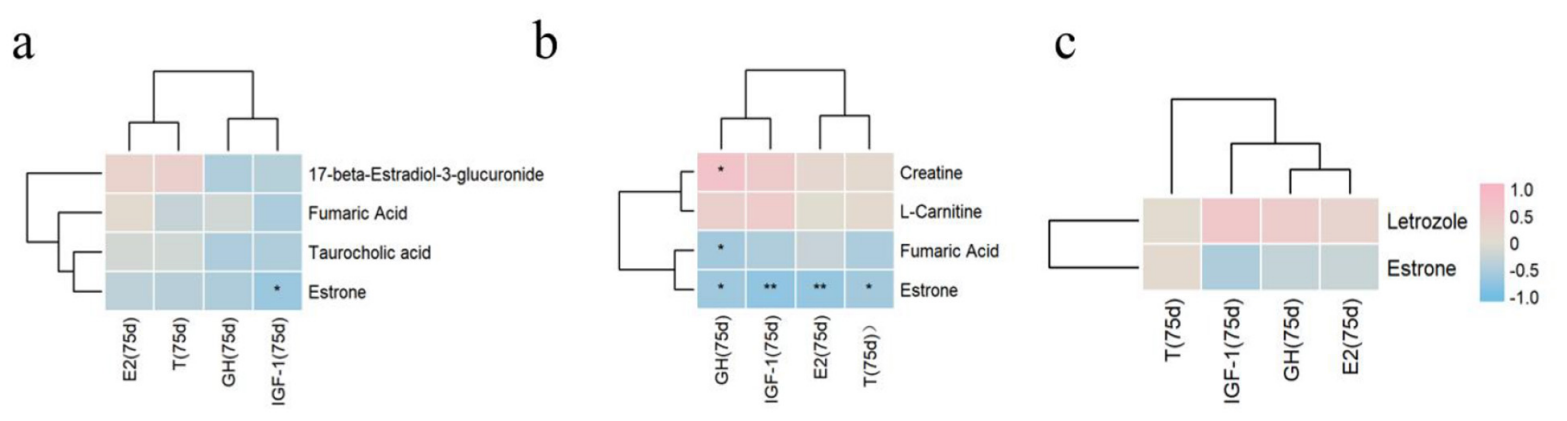
Analysis of plasma metabolites associated with hormones. **(a)** Shows Con group metabolites with differences between urine and plasma levels; **(b)** Shows 0.1 LE group metabolites with differences between urine and plasma levels; **(c)** Shows Con group metabolites with differences between urine and plasma levels. The values on the right represent *P*-values, where *indicates *P* < 0.05, **indicates *P* < 0.01, and ***indicates *P* < 0.001.

As shown in Figure 11a, after 75 days of letrozole supplementation, the Con group exhibited a significant negative correlation with the 0.1 LE group regarding estrone and plasma IGF-1 levels (*P* < 0.05). Compared with 0.2 LE group, the Con group showed a highly significant negative correlation between estrone, IGF-1, and E2 (*P* < 0.01), as well as a significant negative correlation with GH and T (*P* < 0.05; see Figure 11b). There were no significant differences between 0.1 LE group and 0.2 LE group (*P* > 0.05; see Figure 11c).

## 4 Discussion

The juvenile stage represents the period of most rapid growth and development in animals, during which organ functions mature most swiftly. Growth and development during this phase also exert a decisive influence on subsequent production performance. Research indicates that androgens and estrogens not only regulate reproductive performance within the animal reproductive system but also possess the capacity to synergistically regulate growth performance (15). Androgens primarily function by promoting protein synthesis, muscle development, and glucose-lipid metabolism to accelerate growth rates. In contrast, estrogens critically regulate skeletal maturation, terminating linear growth by controlling epiphyseal closure (16). Ma et al. (9) investigated the effects of letrozole on the athletic performance, plasma antioxidant indicators, hormone levels, and body weight of Thoroughbred horses. They found that supplemental letrozole administration increased the body weight of Thoroughbred horses. Chen et al. (17) found that supplementation with different levels of LE increased androgen levels in plasma and seminal plasma while decreasing estrogen levels; in particular, androstenedione and testosterone levels increased significantly. Shen et al. (18) found that LE improved growth performance in yellow catfish 45–75 days post-hatch, indicating its role as an anabolic growth promoter in juveniles. Rezaei et al. (19) demonstrated that injecting goats with 0.25 mg/kg LE significantly increased serum T levels, average daily gain, and carcass weight. In this experiment, both body weight and daily weight gain increased in the experimental group of lambs after supplementation with LE; however, daily weight gain decreased as the supplementation period lengthened. It is speculated that, as the supplementation period extended, the animals may have gradually developed a certain degree of tolerance to LE or a feedback regulatory mechanism, causing its growth-promoting effect to weaken over time.

Following LE supplementation, plasma E2 and T levels in lambs fluctuated significantly, and both GH and IGF-1 levels increased significantly. The study found that the stimulatory effect of elevated androgen levels on GH secretion can accelerate growth in male mice treated with LE (20), consistent with the findings of this study. The primary mechanisms through which IGF-1 exerts its key effects include inhibiting apoptosis and promoting cell proliferation, transformation, and differentiation (21). Research has found that Androgens and Estrogens exert synergistic effects on animal growth performance by regulating GH and IGF-1 through paracrine mechanisms (22). Key target cells along the growth hormone axis express both Androgen Receptor (AR) and Estrogen Receptor (ER). Sex hormones activate similar downstream signaling pathways through distinct receptors. ER enhances GH secretion via the MAPK pathway, while androgens promote animal growth by stimulating AR activity, thereby increasing GH sensitivity and prolonging its effects (23). LE competitively blocks the binding of endogenous substrates to the aromatase active site, reversibly inhibiting aromatase activity. This ultimately specifically suppresses estrogen synthesis by blocking the conversion of AD and T to E2 (24). Under the experimental conditions, weaned female lambs from Turpan Black sheep supplemented with LE exhibited significantly elevated T levels and suppressed E2 synthesis, establishing a dynamic equilibrium between the two hormones. T and E2 synergistically regulate IGF-1 and GH expression levels through paracrine mechanisms. These hormonal changes are presumed to be key factors driving enhanced growth performance in lambs.

In this study, plasma LE concentrations were measured in weaned female Turpan black sheep lambs at different stages of LE supplementation. The results showed that on days 15 and 75 of supplementation, the plasma LE concentration in the 0.2 LE group was highly significantly higher than that in the 0.1 LE group. The expression levels of taurocholic acid, 17β-estradiol-3-β-D-glucuronide, and fumaric acid were upregulated. Estrone is an intermediate product in the conversion of androgens to estrogens and exhibits weaker estrogenic activity than E2. Its upregulation may be associated with the accumulation of androgen precursors resulting from the inhibition of aromatase activity by LE. Taurocholic acid is a primary molecule in the synthesis of bile acids within the bile acid metabolism pathway. It functions to emulsify dietary fats and facilitate vitamin absorption, playing an important role in hepatic metabolism and bile acid metabolism in animals (25). They function to emulsify food and aid in vitamin absorption (26), playing a crucial role in both hepatic metabolism and bile acid metabolism in animals. Previous studies have demonstrated that the estrogen signaling pathway significantly regulates bile acid synthesis and metabolism (27). In this study, supplementation with LE significantly upregulated the metabolites taurochenodeoxycholic acid, taurodeoxycholate, and taurocholic acid. It can be inferred that LE may induce systemic metabolic reprogramming by altering estrogen levels in the body, thereby affecting the regulation of bile acid synthesis enzymes and transporter functions in the liver. This ultimately leads to the accumulation of bile acid metabolites in the bloodstream (28). 17β-Estradiol-3-β-D-glucuronide is a metabolite of 17β-estradiol formed via glucuronidation in the liver. It represents an inactivated form of estrogen and is excreted in the urine (29). LE inhibits estrogen synthesis, leading to a decrease in plasma 17β-estradiol (E2) levels. Consequently, the production of its metabolite, 17β-estradiol-3-β-D-glucuronide, would be expected to decrease. However, in the present study, this metabolite was significantly upregulated in the 0.1 LE group. A possible explanation is that letrozole may activate nuclear receptors such as the pregnane X receptor (PXR) or the constitutive androstane receptor (CAR), thereby inducing the expression of UDP-glucuronosyltransferase (UGT) isoforms (e.g., UGT1A1, UGT2B7) in the liver. Enhanced UGT activity could increase the glucuronidation efficiency, leading to a relative increase in the formation of 17β-estradiol-3-β-D-glucuronide even when the substrate (E2) concentration is reduced (30–32). The exact mechanisms underlying this observation require further investigation, including analyses of the expression and activity of relevant UGT enzymes in liver tissue. Fumaric acid, a key intermediate of the tricarboxylic acid (TCA) cycle and a prosthetic group of the urea cycle, was found to be elevated. This increase suggests that LE supplementation may promote metabolic reprogramming in lambs. A decline in estrogen levels is often accompanied by alterations in lipid metabolism. Notably, fumaric acid can be converted into malic acid by fumarase, thereby participating in the malate-aspartate shuttle and influencing lipid synthesis (33, 34). Compared with the Con group, the 0.2 LE group exhibited upregulation of creatine, and downregulation of estrone, trans-2-hexadecenoyl-*L*-carnitine, and fumaric acid. Studies have shown that creatine supplementation can upregulate the expression of myogenic regulatory factors, increase myofiber diameter, and directly promote muscle growth (35). Upregulation of creatine enhances energy storage in muscle, increases glycogen and ATP levels, and promotes muscle development in lambs (36). This may be associated with the improved growth performance observed in the present study. In addition, LE supplementation reduced estrogen levels in lambs and suppressed the production of estrone, a precursor of estrogen. The trans-2-Hexadecenoyl-L-carnitine is a long-chain acylcarnitine that plays a key role in cellular energy metabolism, particularly in lipid β-oxidation, by transporting long-chain fatty acids into the mitochondrial matrix for oxidative degradation, thereby providing energy for the body (37, 38). Following LE supplementation, the process of lipid catabolism in lambs may be suppressed, and the downregulation of both trans-2-hexadecenoyl-*L*-carnitine and fumaric acid together influences the balance of lipid metabolism, thereby modulating energy partitioning and body fat deposition in lambs. Furthermore, in the comparative analysis of plasma differential metabolites between the 0.1 LE and 0.2 LE groups, letrozole was found to be highly significantly upregulated, and this result was confirmed by a highly significant increase in plasma letrozole concentration measured using HPLC. Collectively, these changes in plasma metabolites represent important features of the metabolic network adjustment in weaned female Turpan black sheep lambs following LE supplementation, providing key clues for a deeper understanding of the metabolic mechanisms through which LE regulates lamb growth and development.

In this study, LE was detected in the rumen fluid of both the 0.1 LE and 0.2 LE groups, and its concentration increased significantly with higher supplementation levels. The ruminal NH_3_-N concentration was significantly elevated in both treatment groups, suggesting that LE may affect the efficiency of nitrogen utilization by rumen microorganisms or protein catabolism, thereby providing a more abundant substrate for microbial protein synthesis and consequently promoting amino acid uptake in lambs. The VFAs are the primary end products of rumen fermentation and serve as a core energy source for ruminants. They also play a role in regulating rumen health, immune homeostasis, and productive performance (39). The increase in propionate helps enhance hepatic gluconeogenesis, providing more utilizable energy for the body (40). Butyrate, as a major energy source for rumen epithelial cells, promotes epithelial development and absorptive function, and helps maintain the intestinal barrier and regulate immune function (41, 42). Under the conditions of this study, the acetate-to-propionate ratio significantly decreased, while butyrate significantly increased, suggesting a shift in rumen fermentation pattern. This shift may be associated with changes in the microbial community and specific metabolic pathways (43). Changes in microbial community abundance may be closely associated with factors such as alterations in the hormonal environment, immune regulation, and enhanced short-chain fatty acid (SCFA) metabolism (44). Firmicutes are primary producers of butyrate and possess a strong ability to degrade cellulose and hemicellulose. Their increased abundance may promote the degradation and absorption of energy substances in feed (45, 46). The *Clostridium_sensu_stricto_1* and *Turicibacter*, both belonging to the phylum Firmicutes, are primary butyrate-producing genera. The short-chain fatty acids they produce can promote osteoblast differentiation and exert bone-protective effects (47). The relative abundances of *s_Butyrivibrio fibrisolvens* in the 0.1 LE group and *g_Oribacterium* in the 0.2 LE group were significantly upregulated. Both taxa belong to the family Lachnospiraceae, whose main fermentation products are butyrate and acetate. Consequently, the relative abundances of the family Lachnospiraceae and its subordinate genus *Lachnospiraceae_NK3A20_group* increased. Succiniclasticum, a genus capable of converting succinate into propionate, plays a key role in energy metabolism in ruminants (48). The *Bacteroidetes* plays a significant role in the degradation and utilization of proteins and carbohydrates and is a major producer of acetate and propionate. An appropriate decrease in its relative abundance leads to a moderate reduction in acetate and propionate levels in the body (49). The *Proteobacteria* and *Fibrobacterota* are also involved in fiber degradation and carbohydrate metabolism (50). It is hypothesized that after LE enters the body, it inhibits estrogen synthesis. As estrogen levels decline, intestinal permeability increases, and the hypoxic environment gradually shifts to a microoxic environment, leading to a weakened anti-inflammatory effect of estrogen and consequently an increase in the relative abundance of *Proteobacteria*. Furthermore, reduced estrogen levels also affect the synthesis and secretion of bile acids. A decrease in bile acids leads to increased viscosity of intestinal contents and reduced fiber digestibility, thereby increasing the relative abundance of *Fibrobacterota* (51). The relative abundance of *Ruminococcus* decreased in both the 0.1 LE and 0.2 LE groups. *Ruminococcus* is a key genus specialized in degrading resistant starch and plant cell walls, and it also participates in acetate synthesis. The decrease in its abundance may indicate that LE supplementation, to some extent, inhibits the degradation of resistant starch and plant cell walls in the rumen. Wang et al. (52) analyzed the gut microbiota of letrozole-induced polycystic ovary syndrome (PCOS) model rats and found an insufficient abundance of bacteria from the genus *Ruminococcus*. This may be attributed to the decrease in estrogen levels and the increase in plasma testosterone following LE supplementation, which leads to reduced bile acid secretion and increased viscosity of intestinal contents, thereby remodeling the intestinal microenvironment and consequently decreasing the relative abundance of *Ruminococcus* (53, 54). The upregulation of the aforementioned microbial taxa may represent an adaptive response of the animal to the altered hormonal environment, helping to maintain rumen microecological homeostasis by enhancing SCFA metabolism, promoting energy uptake, and preserving the intestinal barrier.

Pearson correlation analysis revealed that in the 0.1 LE group, Desulfobulbus showed a significant negative correlation with 17β-estradiol-3-β-D-glucuronide, and tended to be positively correlated with acetate, propionate, and butyrate. Studies have shown that *Desulfobulbus* can oxidize propionate to acetate and CO_2_ in the presence of sulfate, and convert lactate, pyruvate, or ethanol to propionate and acetate under fermentative conditions (55). Therefore, when the relative abundance of *Desulfobulbus* increases, the levels of acetate and propionate are correspondingly upregulated. Kumari et al. (56) identified a set of genes in the gut microbiota capable of modulating estrogen metabolism, with β-glucuronidase being a key enzyme. This enzyme influences the gut microbiota and affects estradiol metabolism. Specifically, β-glucuronidase hydrolyzes 17β-estradiol-3-β-D-glucuronide, reconverting it into free estrone (57, 58). While the microbiota metabolizes hormones, hormones may also directly act as signaling molecules to influence microbial growth (59). However, no direct association between *Desulfobulbus* and the metabolism of 17β-estradiol-3-β-D-glucuronide has been reported to date, and the specific mechanisms remain to be further elucidated. As the supplemental dose of letrozole increased, plasma estrone levels decreased significantly. Ruminococcus is also one of the key genera producing β-glucuronidase, an enzyme responsible for the enterohepatic circulation of estrogens (60). Previous studies have shown that E2 combined with antibody treatment can reshape the gut microbiota structure, and that E2-treated mice exhibited a significant increase in the relative abundance of *Ruminococcus* (61). In the present study, Ruminococcus showed a significant negative correlation with butyrate and a positive (non-significant) trend with isovalerate. Niu et al. (62) reported that increased abundance of *Ruminococcus* in the rumen was accompanied by a significant decrease in butyrate concentration and a significant increase in isovalerate concentration. Zhang et al. (63) found a significant negative correlation between *Ruminococcus* and butyrate in the rumen of finishing lambs. *Ruminococcus* is a producer of isovalerate, and its abundance directly determines isovalerate content (64). Early studies have shown that GH can directly promote creatine synthesis and accumulation in muscles, thereby supporting high-energy phosphate supply, protein deposition, and rapid growth (65). Under the conditions of the present study, creatine was significantly positively correlated with GH. GH is a core hormone regulating animal growth, development, and metabolism, and its synthesis and secretion involve multiple levels of regulatory mechanisms (66). GH also regulates ovarian steroid hormone production by binding to growth hormone receptors (GHR) on the ovary, improving ovarian responsiveness to gonadotropins (Gn) and promoting follicular growth, development, and maturation (67). The LE inhibits the conversion of androgens to estrogens, leading to a decrease in estrogen levels. This reduction attenuates the negative feedback inhibition on the hypothalamic-pituitary-growth axis, thereby promoting the upregulation of growth hormone (GH) secretion. However, as the supplemental dose of LE increased, plasma estrone levels decreased, and *Clostridium_sensu_stricto_1, Romboutsia*, and *Turicibacter* showed negative correlations with LE concentration and positive correlations with estrone. It is speculated that this result is dose-dependent: with increasing letrozole dosage, the growth and proliferation of these genera may partially rely on a certain level of estrogenic environment, and thus their abundances decline as estrone levels fall. Furthermore, long-term supplementation with a high dose of letrozole may indirectly affect the rumen microenvironment, altering the rumen fermentation pattern and inducing mismatch repair and homologous recombination in the microbiota, which in turn influences the colonization and metabolic activity of the rumen microbiota, resulting in the observed negative correlations with letrozole concentration. The specific mechanisms underlying these effects require further investigation and validation.

## 5 Conclusion

**Figure 12:**
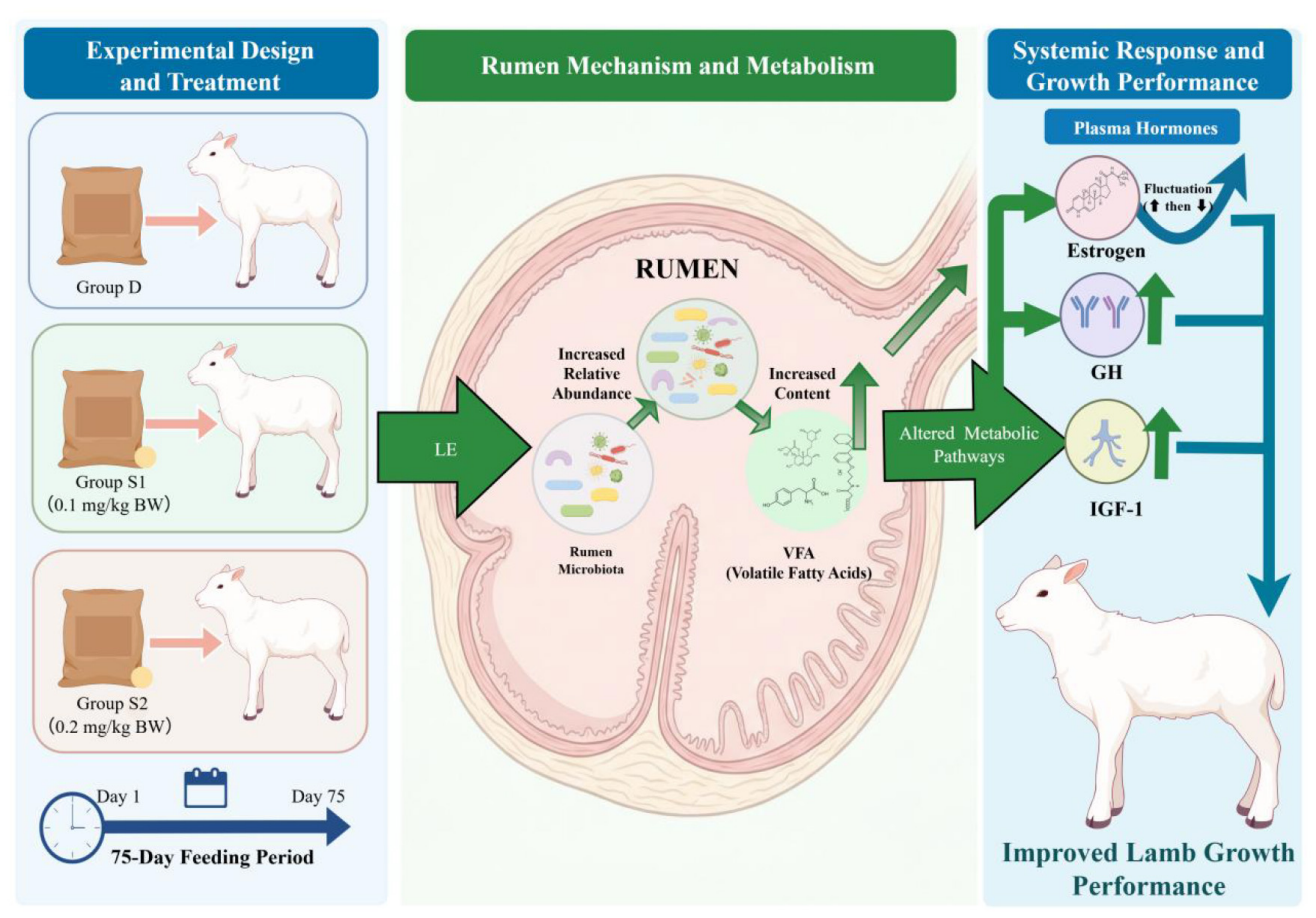
Results of the effect of supplemental LE feeding on lamb growth performance.

Dietary letrozole (LE) supplementation effectively shifted rumen fermentation from an acetogenic to a propionic and butyric acid pattern in fattening lambs, thereby improving feed utilization. LE also modulated plasma metabolites involved in bile acid and tryptophan pathways, leading to changes in estrogen, GH, and IGF-1 levels that supported growth. Among the doses tested, 0.20 mg/kg BW^−1^ during a 75-day finishing period for 2-month-old weaned female lambs produced the best outcomes. These findings provide a practical basis for using LE as a rumen fermentation modulator to enhance production efficiency in sheep (Figure 12).

## 6 Limitations

As a preliminary exploratory study, this trial still has room for expansion in terms of both depth and scope. The sample size was determined based on standard feeding trial protocols; in the next phase, the sample size should be appropriately increased and the observation period extended, which will better facilitate the understanding of letrozole's long-term effects and underlying patterns. This study did not fully account for letrozole's half-life; consequently, the blood sampling protocol at different time points was not sufficiently refined and failed to accurately reflect the dynamic changes of letrozole in the body.

## Data Availability

The datasets presented in this study can be found in online repositories. NCBI, accession number: PRJNA1431764, https://www.ncbi.nlm.nih.gov/sra/PRJNA1431764.
